# Can introvoxel incoherent motion MRI be used to differentiate patients with placenta accreta spectrum disorders?

**DOI:** 10.1186/s12884-019-2676-x

**Published:** 2019-12-30

**Authors:** Tao Lu, Hong Pu, Kui-de Li, Jie Mei, Meng-wei Huang, Shao-yu Wang

**Affiliations:** 1 Department of Radiology, Sichuan Academy of Medical Sciences & Sichuan Provincial People’s Hospital, 32 West Second Section, First Ring Road, Chengdu, 610072 Sichuan China; 2 Department of Obstetrics, Sichuan Academy of Medical Sciences & Sichuan Provincial People’s Hospital, 32 West Second Section, First Ring Road, Chengdu, 610072 Sichuan China; 3 MR Scientific Marketing Specialist, Siemens Healthineer, No.278, Zhouzhu Road, Pudong New Area District, Shanghai, 201318 China

**Keywords:** PAS disorders MRI placenta IVIM

## Abstract

**Background:**

The incidence of PAS disorders increased rapidly in recent years, and introvoxel incoherent motion (IVIM) MRI has been applied in the assessment of placenta. The study aims to investigate whether the parameters from IVIM can be used to differentiate patients with PAS disorders complicating placenta previa and secondly to differentiate different categories of PAS disorders.

**Methods:**

The study participants were comprised of 99 patients with placenta previa, including 16 patients with placenta accreta, 51 patients with increta, 8 patients with percreta and 24 patients without PAS disorders between 28 + 0 and 39 + 6 weeks. IVIM MRI was performed on a 1.5 T scanner. Perfusion fraction (f), pseudodiffusion coefficient (D*) and diffusion coefficient (D) were calculated.

**Results:**

Women with PAS disorders had a higher perfusion fraction (*p* = 0.019) than women without the disease. Multiple comparisons showed perfusion fraction in patients without PAS disorders was significantly lower than in patients with placenta accreta and percreta(*P* = 0.018 and 0.033 respectively), but was not lower than in patients with increta(*p* = 1).

**Conclusion:**

Patients with placenta accreta and percreta differed in placental perfusion fraction from women with increta and without PAS disorders.

## Background

The term placenta accreta spectrum disorders was adopted by FIGO recently, it covers a spectrum disorders from abnormal adherence (placenta accreta) to abnormal invasion (placenta increta and percreta). The incidence of PAS increased rapidly in recent years, from1 in 533 pregnancies for the period of 1982–2002, to 1 in 4027 pregnancies in the 1970s and 1 in 2510 pregnancies in the 1980s [1, 2]. Placenta previa and previous cesarean section represent the two major risk factors for PAS disorders.

Prenatal assessment of placental perfusion would be of considerable value for the diagnosis of PAS disorders and allows for delivery planning in an attempt to reduce delivery complications and predict clinical outcome. MRI is increasingly widely used in modern obstetrics and benefits from the experience of functional MRI, which allows the assessment of placental vascular physiology and function. IVIM is a noninvasive in vivo measurement which offers a quantitative technique to measure maternal placental perfusion and no contrast agent is used. Applications of IVIM were mainly in the liver, pancreas, kidney and prostate analysis currently [3–8]. Owing to the highly vascularized characteristic of the placenta with both a high blood fraction and a large perfusion component, it is also appropriate for evaluation with IVIM.

IVIM was adopted in a few small previous studies including placenta perfusion in normal pregnancies, pregnancies affected by intrauterine growth restriction and preeclampsia-complicated pregnancies [9–12]. Using IVIM, Jakab et al. found perfusion fraction of the placenta moderately increased during gestation and correlated negatively with the umbilical artery resistance index [9]. Moore et al. identified different zones of blood movement were visible within the placentas in normal pregnancies, whereas the placentas appeared far more homogeneous in IUGR-complicated pregnancies, with their outer zones containing a significantly reduced proportion of moving blood compared to the normal cases [10, 11]. Sohlberg et al. found a smaller placenta perfusion fraction in women with early preeclampsia (< 34 weeks) and a larger one in women with late preeclampsia (> 34 weeks) when was compared in women with normal pregnancies, secondly, they found placental perfusion fraction decreased with increasing gestational age in normal pregnancies [12]. Melbourne et al. developed a combined model consisting both IVIM and T2 relaxometry and generated new predictive measurements which would be of help in the understanding of the physiological properties of the placenta [13].

In a preliminary study, we found a decreased perfusion fraction in women with placenta accreta and increta [14]. But we did not include patients with placenta percreta nor explored the diagnostic value of the perfusion fraction. Thus, our purpose was to investigate whether the parameters from IVIM could be used to differentiate patients with PAS disorders complicating placenta previa and to differentiate different categories of PAS disorders.

## Methods

**Fig. 1:**
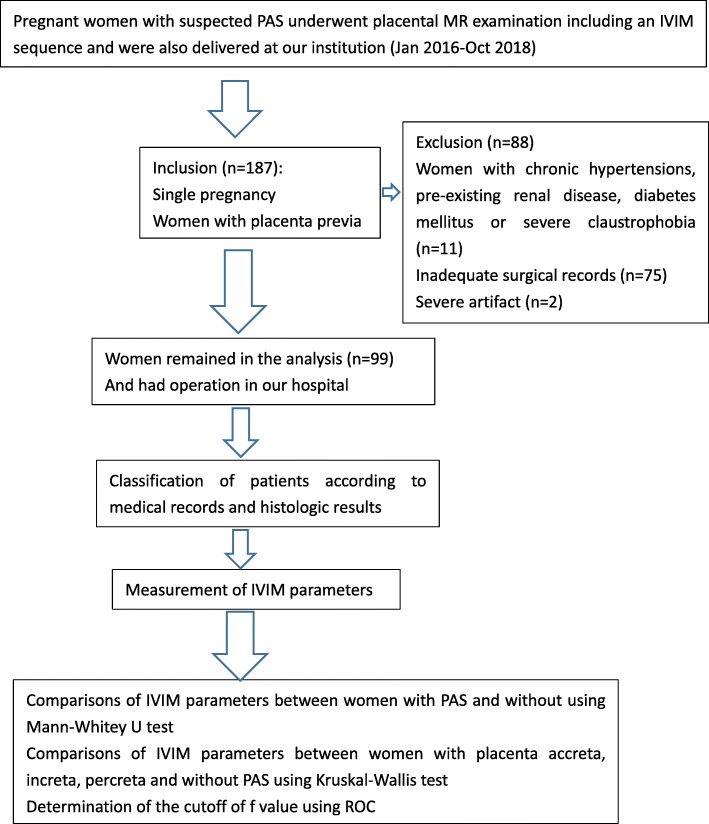
Flowchart of the study design

The ethical review aboard of our hospital approved the study and informed consent was obtained from each woman participating in the study. Between Jan 2016 and Oct 2018, 206 gravid patients in the third trimester of pregnancy were referred for prenatal MRI, dedicated to placental evaluation; Women with placenta previa (*n* = 187), single pregnancy with a living fetus and a gestation length between 28 + 0 and 39 + 6 weeks were included. All the pregnancies were dated by ultrasound scan in the first and second trimester. Women with chronic hypertensions, pre-existing renal disease, diabetes mellitus (*n* = 11) were excluded. Women with inadequate surgical records (*n* = 75) or severe artifact (*n* = 2) were also excluded. 99 patients completed the MR examination and formed our study group (Fig. 1). The specific tomography of placental invasion was established in the operating room according to clinical and anatomical criterion. The final degree of placental invasion was established either by placental villi alterations from placental sample or from maternity records of the women’s general practitioners. For patients who had total or subtotal hysterectomy, histologic criteria for PAS were based on chorionic villi attachment to the myometrium. For patients who did not require hysterectomy, the final diagnostic criteria of placental invasion used in this study included the following: during CD, placenta accreta was defined as the placenta adhered firmly to the endometrium without invasion and showed non-self-controlled bleeding when detached, placenta increta was defined as the placenta deeply implanted in the myometrium and required curettage to remove invasive tissue, and placenta percreta was defined as the placenta villi penetrated through the uterine serosa or the surrounding anatomical structures.

### Imaging techniques

MR imaging was performed on a 1.5 T scanner (Magnetom Area, Siemens, Erlanger, Germany) using the integrated whole-body transmit-receive coil. IVIM sequence was used acquisition matrix 192 × 120, FOV 390 × 304 mm, slice thickness 5.5 mm, number of slices 40, and 8 different b-values (0,50,100,150,200,250,500,800 s/m2) perpendicular to the placenta. Acquisition time for this sequence was typically 8 min and 37 s.

### Region of interest

**Fig. 2:**
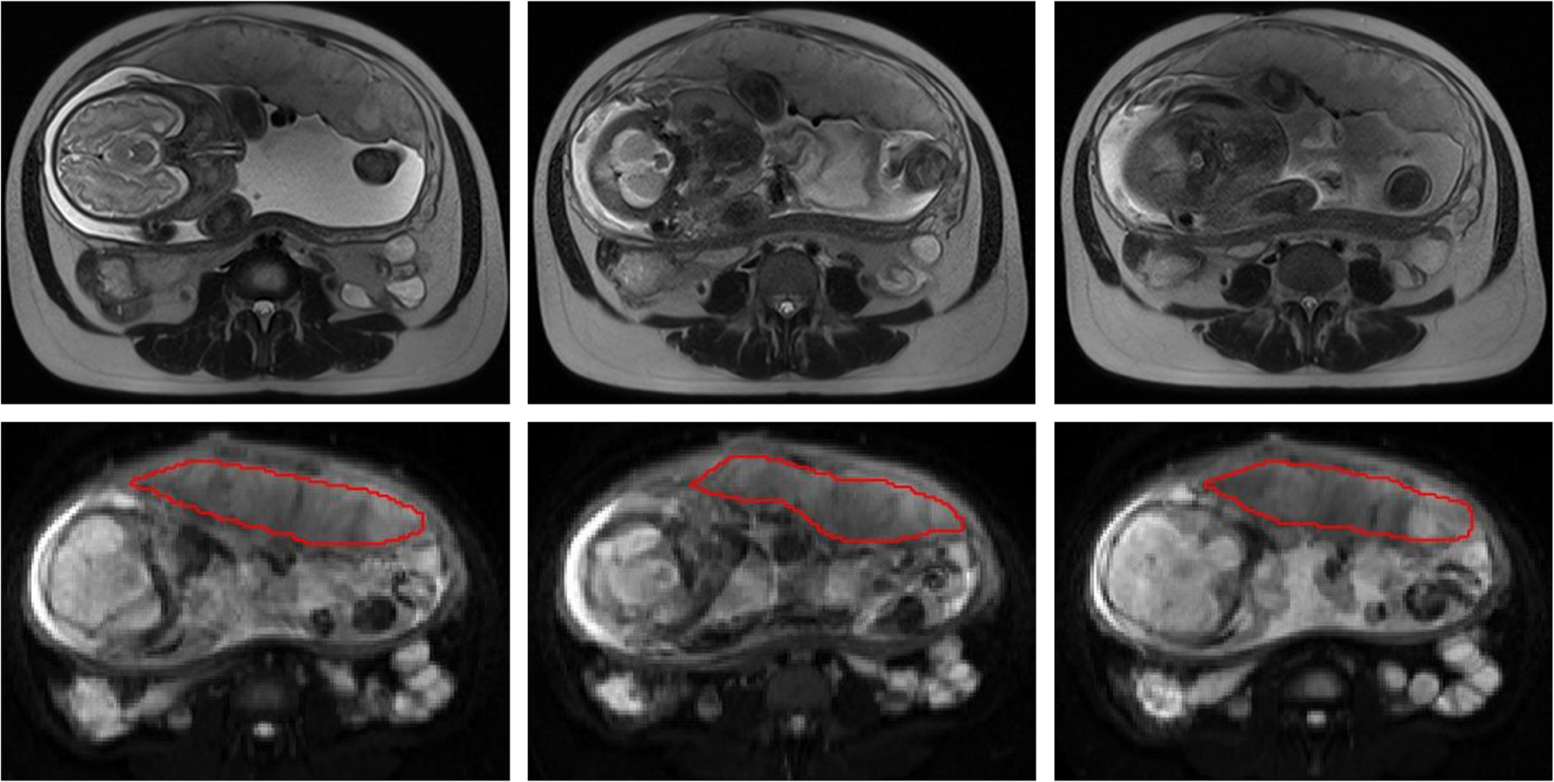
ROI of the placenta

Images obviously affected by subject motion were discarded from the analysis. Evaluation of the IVIM sequence was performed with research software (MITK diffusion). Two independent blinded observers, with 3 and 10 years of experience in obstetric imaging, respectively, carried out the measurement of IVIM. In patients without PAS disorders, ROIs were placed in the middle part of the placenta including as large parts of the placenta as possible, but excluding areas with infarcts, hemorrhage or other artifactual signal loss (Fig. 2). The same ROIs were draw on the slice above and below the middle slice. In patients with PAS disorders, the ROIs were placed in the regions of placental adhesion according to the maternity record after surgery, and the same ROIs were draw on the slice above and below the adhesion slice. At least 2 cm from the insertion of the umbilical cord should be adopted for the ROIs to avoid the flow artifacts in large vessels. We calculated the values of D, D* and f by averaging over 3 ROIs totally.

### Statistics

Inter-rater reliability of the f, D, D* values between 2 radiologists was assessed by the intra-class correlation coefficient based on a mean-rating (k = 2), absolute-agreement,2-way random model. Physical and sociodemographic factors for PAS disorders were investigated by using the chi-square, student’s t-test or the Mann-Whitney u test to compare patients with PAS disorders to those without. The IVIM parameters were compared using Mann-Whitney u test or Kruskal-Wallis test. Receiver operating characteristic curve (ROC) was used to determine the cutoff of f value with the best sensitivity and specificity for distinguishing patients with PAS disorders from patients without the disease. *P* values< 0.05 were considered statistically significant. All analyses were performed using IBM SPSS statistics 20.

## Results

Ninety-nine pregnant women with satisfied raw images remained in the analysis (Fig. 2). Intra-class correlation coefficient between 2 radiologists of f, D and D* values were 0.892, 0.775 and 0.541 respectively. The mean maternal age was 31.92 ± 4.31 years(range22–41 years), the mean gestational age at examination was 34(4) weeks(range 28 – 39 weeks). All gravid patients had placenta previa (complete, *n* = 70; marginal, *n* = 22; partial, *n* = 4; low lying, *n* = 3).

All medical records were received postpartum, 16 patients were diagnosed as placenta accreta when the placenta attaches onto the myometrium, 51 patients were diagnosed as placenta increta when the placenta penetrated into the myometrium, 8 patients were diagnosed as placenta percreta when the placenta penetrated through the uterine serosa, and 24 patients were without PAS disorders. The methods of delivery included 50 cases of cesarean section, 45 cases of prophylactic distal abdominal aorta balloon occlusion and cesarean section, 2 cases of natural birth, 2 cases of total hysterectomy and 1 case of partial hysterectomy.

**Fig. 3:**
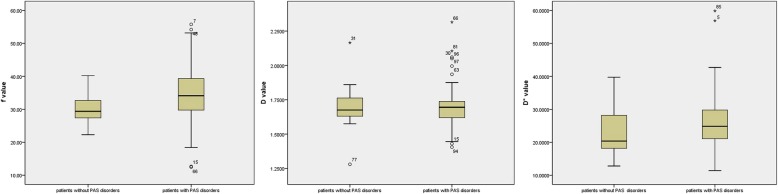
variation of f value. Box plot represented the distribution of f value in patients without PAS and with PAS

**Fig. 4:**
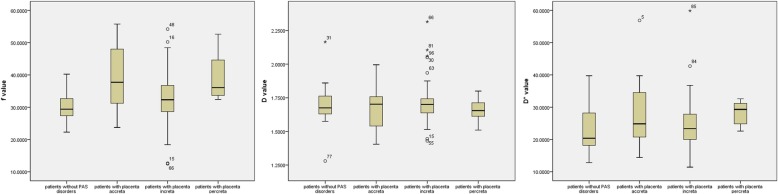
variation of f value. Box plot represented the distribution of f value in patients with placenta accreta, increta, percreta and without PAS

Table 1 presents the maternal characteristics of the study participants. Numbers of previous cesarean section was greater in patients with PAS disorders than in patients without the disease (*p* = 0.021). The blood loss during delivery in patients with PAS disorders was greater (*p* = 0.001), and more patients with PAS disorders required transfusion (*p* = 0.003). When compared with patients with PAS disorders, f and D* values were significantly lower in patients without the disease (*p* = 0.019and0.044, respectively), while D values showed no statistical significance between the 2 groups (*p* = 0.915). Fig. 3 shows a comparison of the parameters between the 2 groups. Based on the receiver operating characteristic curve analysis, the area under the curve was o.659 with a sensitivity of 64% and specificity of 71%, the best cutoff of f value was 0.32. Multiple comparisons showed f values in patients without PAS disorders were significantly lower than f values in patients with placenta accreta and percreta (*p* = 0.018 and 0.033 respectively), but were not significantly lower than in patients with increta(*p* = 1). Difference from the 4 groups in D and D* values was not statistically significant (Table 2). Fig. 4 shows a comparison of the parameters between the 4 groups.

**Table 1:** Physical and sociodemographic features of patients studied

	Patients without PAS disoders	Patients with PAS disoders	*P* value
Number	24 (24.24%)	75 (75.76%)	
Age (years)	31.88 ± 3.81	31.93 ± 4.48	0.147
Less than 35	20 (20.20%)	51 (51.52%)	
35 or older	4 (4.04%)	24 (24.24%)	
Gestational age At examination (weeks)	33.5 (4)	34 (4)	0.825
Gestational age At the time of delivery (weeks)	37 (2)	37 (1)	0.241
Number of Previous caesarean Section			
0	11 (11.11%)	15 (15.15%)	
1	12 (12.12%)	46 (46.46%)	
2 or more	1 (1.01%)	14 (14.14%)	0.021
Previous uterine Dilation and Curettage			
No	2 (2.02%)	16 (16.16%)	
Yes	22 (22.22%)	59 (59.60%)	0.226
Blood lost (ml)	500 (200)	800 (600)	0.001
Transfusion	6 (13.64%)	12 (27.27%)	0.003
No	21 (21.21%)	40 (40.40%)	
Yes	3 (3.03%)	35 (35.35%)	
Amount of transfusion (ml)	0 (0)	0 (800)	0.002
f (%)	29.39 (5.59)	34.12 (9.8)	0.019
D (10^−3^ mm^2^/s)	1.68 (0.13)	1.7 (0.13)	0.915
D*(10^−3^ mm^2^/s)	20.37 (10.22)	24.84 (8.94)	0.044

**Table 2:** Multiple comparsion between different groups of three parameters (*n* = 44)

Group	n	f (%)	D(10^− 3^ mm^2^/s)	D*(10^− 3^ mm^2^/s)
Patients without PAS disorders	24 (24.24%)	29.39 (5.59)	1.68 (0.13)	20.37 (10.22)
Patients with Placenta accreta	16 (16.16%)	37.73 (18.17)	1.70 (0.23)	24.84 (14.35)
Patients with Placenta increta	51 (51.52%)	32.32 (8.56)	1.70 (0.12)	23.35 (9.19)
Patients with Placenta percreta	8 (8.08)	36.01 (13.27)	1.66 (0.11)	29.31 (7.26)
*P* value		0.016	0.794	0.160

## Discussion

The most commonly described risk factors for PAS disorders were previous cesarean delivery and placenta previa. Placenta previa in particular has been shown to be a significant risk factor for PAS disorders, associated with a 9.3% incidence of abnormal placentation in one series, with higher risk in women have undergone prior cesarean deliveries [15]. According to Yu’s report, the incidence of placenta previa was 10.96% in one center in China, the average incidence of placenta accreta in patients with both prior cesarean delivery and placenta previa was 2.08‰ [16]. In our study, the study participants were patients with placenta previa and were suspected of having PAS disorders from results from ultrasonography. More patients with PAS disorders had previous cesarean deliveries, which was in accordance with the previous reports.

Functional MRI and radiomics from MRI images are now being introduced in pregnancy. Texture analysis is a part of radiomics and it can achieve quantitative measurement of heterogeneity and other features from images which are beyond the visibility of the eyes [17]. In a recent study of texture analysis of placenta MRI, Chen et al. [18] found PAS was associated with higher values for standard deviation of pixel intensity and fractal analysis at every box size which suggested a potential role of the texture analysis in helping the diagnosis of PAS. With the help of functional MRI, we can acquire both morphological and physiological information of the placenta and potentially improves diagnosis of pregnancy complications. DWI is already commonly used for fetal imaging, and IVIM is a derivative of DWI that describes the movement of blood within a single imaging voxel based on a bi-exponential model. IVIM is sensitive for organs with a high blood fraction, so it is appropriate for the evaluation of the placenta. It has already been used in pregnancies complicated by PE and IUGR in recent years. In the previous study using IVIM, we found a decreased perfusion fraction in patients with placenta accreta and increta. However, the exact physiologic alterations of the placental perfusion in patients with PAS disorders have not been fully understood.

Before a parameter can be put into clinical use, we need to ensure the reliability in repeated measurement with acceptable reproducibility, especially when considering the potential artifacts caused by respiratory or cardiac motion [19–21]. The reproducibility of f and D values was good when scanning with the IVIM sequence in our study. The results showed that f and D had excellent interobserver agreement, while D* had poor interobserver agreement which was similar to previous findings. The intrinsic inhomogeneous perfusion alteration, low signal-to-noise ratio in abdominal DWI, and the limitation in current nonlinear least square fitting method maybe attribute to the worst interobserver agreement of D* [21, 22]..

The diffusion coefficient reflects cellular and interstitial characteristics of the tissue. Our study showed that D value did not differ between patients with PAS disorders and patients without the disease. This maybe because tissue diffusion did not change in the region where the placenta adhered to the myometrium abnormally.

In our study, more patients with PAS disorders had previous cesarean deliveries, f value was also significantly higher in patients with PAS disorders. Uterine scarring from previous cesarean deliveries can cause local decidual deficit, then leading to invasive placentation. Histological studies demonstrated the number of partially or non-remodeled spiral arteries in placenta accreta increased even in the presence of abundant extravillous trophoblast, accompanying abnormal EVT invasion into radial and arcuate arteries deep within the myometrium [23, 24]. These changes may explain the hypervascular nature of the placental bed in abnormally invasive placenta, which was also verified by a dynamic enhanced MRI study of the placenta [25]. So the perfusion fraction increased in patients with PAS disorders. Secondly, we expected to find a best cutoff of f value to differentiate patients with PAS disorders from patients without the disease. The area under the curve was just 0.659 with a sensitivity of 64% and a specificity of 71%.

To explore the reason, we further divided patients with PAS disorders into patients with placenta accreta, patients with increta and patients with percreta. We found an increase of perfusion fraction in patients with placenta accreta, then a slight decrease in patients with increta, and last another increase in patients with percreta. The f values were significantly lower in patients without PAS disorders than in patients with placenta accreta and percreta, but were not significantly lower than in patients with increta. Pathologically, in placenta accreta, the placenta villi embedded directly onto the myometrium in the absence of the decidua [26]. We assumed that the spiral arterial remodeling is only mildly reduced, while more maternal blood in the myometrium bathe the fetal villi, so the perfusion fraction increased in placenta accreta. In placenta increta, the chorionic villi are found deeper into the myometrium, the spiral arteries remodeling is further reduced [27], the placental perfusion is thus balanced in this stage. So the perfusion fraction is similar to that in normal placenta. In placenta percreta, the placenta villi penetrate the uterine serosa. Although spiral arteries remodeling persists to reduce, in conjunction with vessel wall infiltration of larger arteries of the radial, arcuate system, numbers of small vessels close to the placental-myometrium junction increase deep to the myometrium [23], so the perfusion fraction increased again in placenta percreta. In consequence, it’s imprudent to simply use perfusion fraction to differentiate patients with PAS disorders from patients without the disease. Since the perfusion fraction did not show statistical difference between patients with placenta increta and patients without PAS disorders, it is possible to confuse the two entities solely rely on perfusion fraction.

The study has several limitations. First, this is a retrospective study, not a prospective one. We mainly measured the areas of placenta invasion according to the description of maternity record instead of measurement of the entire placenta. From our previous experience, IVIM parameters did not show statistical difference between the site where placenta separated from uterine wall normally in the patients with placenta accreta and the regions of placenta without placenta accreta [14]. Chen et al. [18] also argued that instead of the entire placenta, future studies can focus on only abnormally high risk areas adjacent to the cesarean scar, then more predictive results may be produced, as more homogeneous regions adjacent to abnormal areas may skew the data. Second, our study population mainly included patients with high risk of PAS disorders after ultrasonography, so the results may be biased as MRI is not a method used for screening. Third, it is possible that the power of statistical analysis is limited since the number of patients in this study is small. However, our study could serve as a preliminary finding for further studies with larger sample sizes. Fourth, We acknowledged the biexponential fitting of IVIM was very sensitive to artifacts including respiratory motion, bowel movement, field inhomogeneity, and magnetic susceptibility artifacts, et al. So the parameters of IVIM may be influenced in this regard. Faster techniques and techniques less prone to signal loss from differences in magnetic susceptibility at air-tissue interfaces, as well as image registration technique to correct for motion prior to IVIM analysis may help reduce this problem [12, 28].

## Conclusions

Perfusion fraction can be used to quantitatively assess placental perfusion in patients with PAS disorders. The perfusion fraction increased in patients with PAS disorders complicating placenta previa, mainly in patients with placenta accreta and percreta.

## Data Availability

Data supporting the results reported in the article can be found in the PACS system of Sichuan Provincial People’s Hospital.

## Abbreviations

CD: Cesarean delivery
DWI: Diffusion weighted imaging
EVT: Extravillous trophoblast
FIGO: International federation of gynecology and obstetrics
IUGR: Intrauterine growth restriction
IVIM: Introvoxel incoherent motion
MRI: Magnetic resonance imaging
PAS: Placenta accreta spectrum
PE: Preeclampsia
ROI: Region of interests
